# Using Event-Related Brain Potentials to Assess Perceptibility: The Case of French Speakers and English [h]

**DOI:** 10.3389/fpsyg.2016.01469

**Published:** 2016-10-04

**Authors:** Jennifer Mah, Heather Goad, Karsten Steinhauer

**Affiliations:** ^1^Department of Humanities, Faculty of Arts, Mount Royal UniversityCalgary, AB, Canada; ^2^Department of Linguistics, McGill UniversityMontreal, QC, Canada; ^3^Language Research Centre, University of CalgaryCalgary, AB, Canada; ^4^Centre for Research on Brain, Language and Music, McGill UniversityMontreal, QC, Canada; ^5^Neurocognition of Language Laboratory, School of Communication Sciences and Disorders, Faculty of Medicine, McGill UniversityMontreal, QC, Canada

**Keywords:** phoneme perception, second language acquisition, ERP, MMN, [h], laryngeals, English, French

## Abstract

French speaking learners of English encounter persistent difficulty acquiring English [h], thus confusing words like *eat* and *heat* in both production and perception. We assess the hypothesis that the acoustic properties of [h] may render detection of this segment in the speech stream insufficiently reliable for second language acquisition. We use the mismatch negativity (MMN) in event-related potentials to investigate [h] perception in French speaking learners of English and native English controls, comparing both linguistic and non-linguistic conditions in an unattended oddball paradigm. Unlike native speakers, French learners of English elicit an MMN response only in the non-linguistic condition. Our results provide neurobiological evidence against the hypothesis that French speakers’ difficulties with [h] are acoustically based. They instead suggest that the problem is in constructing an appropriate phonological representation for [h] in the interlanguage grammar.

## Introduction

In acquiring English as a second language, native speakers of French have been observed to encounter persistent difficulty with [h], a sound that is absent from the phonetic and phonemic inventories of French. In production, patterns of both deletion of [h] from [h]-initial words and inappropriate epenthesis of [h] onto vowel-initial words have been reported (Janda and Auger, 1992; John, 2006). In perception, French speakers who were very advanced English learners with training in English phonetics performed significantly worse than native speaker controls in discrimination of [h] vs. Ø pairs (e.g., *heat* vs. *eat*; LaCharité and Prévost, 1999). While a recent study by White et al. (2015) suggests that attentional factors may have a role to play in successful discrimination of [h], it remains unclear what underlies French speakers’ errors, and why difficulties with [h] can persist, even among very advanced learners. Part of the problem may lie in the fact that acquiring [h], unlike acquiring other consonants, does not involve learning to distinguish it from another consonant; rather, it involves learning to distinguish it from silence.

This paper aims to experimentally identify the root cause of difficulties that French-speaking learners have with English [h]. A plausible hypothesis is that these difficulties are due to the acoustic properties of [h], which make it perceptually weak. Consequently, French speakers may be unable to reliably detect [h] in the speech stream. We present neurobiological evidence from event-related brain potentials (ERPs) against this hypothesis, indicating that the difficulty for these learners lies in the phonological representation of [h], and not in this segment’s acoustic properties. We begin by discussing the exceptional phonetic properties of [h]. We then exemplify the distributional patterns that [h] displays when compared with other consonants in English.

### The Phonetic Properties of [h]

Phonetically, laryngeals ([h] and glottal stop) are not like other consonants in that they are produced at the larynx, with no appreciable constriction in the oral and pharyngeal cavities (McCarthy, 1994). For [h], the vocal folds are abducted; the absence of vocal fold vibration results in voicelessness. Continuous airflow is maintained throughout production of the segment, but the absence of constriction in the oral or pharyngeal cavities means that the airflow never becomes turbulent as it passes through the vocal tract. Generally, fricatives are characterized by the turbulence produced at a given place of articulation, the location where the airflow is constricted in the oral cavity (Ladefoged, 2001). For example, high intensity [s] results from forcing air through a narrow channel, with its narrowest point at the alveolar ridge; air passes through this narrowing and strikes the teeth, producing high-frequency turbulence. Conversely, low intensity [𝜃] (as in *thumb*) results from forcing air through a wider channel; although the airflow strikes the teeth, it is not with the same force due to increased constriction width (Narayanan et al., 1995). The absence of supralaryngeal narrowing in the production of [h] results in particularly low intensity fricative noise. As [h] lacks the turbulent airflow that characterizes other fricatives, it is more accurately described as the voiceless counterpart of an adjacent vowel (Ladefoged and Maddieson, 1996), with air flowing through a relatively open vocal tract, encountering no obstacles to create turbulence. These articulatory properties conspire, with the result being that [h] is perceptually weak, and thus difficult to detect in the speech stream.

### Phonological Consequences of the Phonetic Properties of [h]

The phonetic properties of [h] parallel this segment’s phonological representation. The absence of supralaryngeal constriction has led to the proposal that [h] lacks place features (e.g., Steriade, 1987; Rose, 1996); the absence of turbulent airflow suggests that it has no manner features (Dogil, 1988; McCarthy, 1988). This highly impoverished representation reflects the fact that [h] displays behavior not observed for other consonants.

In English, for example, [h] only appears word-initially and at the beginning of stressed syllables: [h]*o.rí.zon* (cf. *bráħ*.*min*), *ve.*[h]*í.cu.lar* (cf. *vé*.*ħi*.*cle*) (ħ indicates non-realization of [h]; periods mark syllable boundaries; Davis and Cho, 2003). By contrast, [𝜃] is not subject to such distributional restrictions: [𝜃]*ó*.*rough*, *á*[𝜃].*lete*, *me*.[𝜃]*ó*.*di*.*cal*, *mé*.[𝜃]*od*. The observation is that [h] is restricted to positions where its audibility is maximized (e.g., stressed syllables have greater amplitude and duration compared to unstressed syllables).

### Current Study

While the low perceptual salience of [h] is augmented by this segment’s distribution in English, the observation that French speakers encounter persistent difficulty with this segment suggests that this enhancement may be insufficient for second language learners whose native language lacks [h] altogether. Recall that Janda and Auger (1992) found patterns of [h]-deletion from [h]-initial words (e.g., *‘elp* for ‘help’) and [h]-epenthesis on vowel-initial words (e.g., *[h]as* for ‘as’), sometimes both occurring within a single word (e.g., *‘ead[h]ache* for ‘headache’).^1^ Importantly, Janda and Auger’s (1992) data were drawn from spontaneous speech samples produced by French speaking learners of English (henceforth: ‘learners’) who had been living in an English-speaking environment for many years, described themselves as advanced English speakers, and used English in their day-to-day lives. Similarly, the participants in LaCharité and Prévost’s (1999) perception study were described as very advanced learners who had completed a course in English phonetics and were preparing for careers as English teachers. Still, these individuals performed significantly worse than native English controls on an AX discrimination task involving [h] vs. Ø: when presented with pairs of words, the learners made more errors than the controls in identifying [h] vs. Ø pairs as either ‘same’ (e.g., *heat* vs. *heat*) or ‘different’ (e.g., *heat* vs. *eat*). By contrast, these same learners performed as well as the controls on [t] vs. [𝜃]; like [h], [𝜃] is also absent from French, suggesting that learners find English [h] problematic because they cannot reliably detect it in the speech stream, and therefore cannot construct an appropriate representation for it in the grammar. Essentially, even though the distribution of [h] in English affords them the best chances of hearing it, the low perceptibility of this segment cannot be overcome.

If the observed difficulties with English [h] are due to this segment’s acoustic properties, then learners should find the segment equally difficult to detect whether it is perceived as part of a *linguistic* speech stream or not. To test this, we employed the experimental design of Werker and Tees (1984). These researchers demonstrated that adults are better able to discriminate segmental contrasts not found in their native language when they are presented in a manner where they would not be identified as linguistic data. Native speakers of English performed poorly in discriminating the Thompson Salish plain [q] vs. ejective uvular [q’] contrast when these were presented in CV syllables, but these same speakers performed well when the syllables were truncated to remove the vowel portion, leaving only the noise burst of the stop release, which resembled clicks more than they did any language the participants were familiar with.

The current study seeks to test the possibility that learners’ persistent difficulties with English [h] reflect a general problem perceiving the acoustic signal associated with [h], due to its non-salience, rather than a localized problem perceiving the acoustic signal as a linguistic event. That is, we examine the possibility that the difficulty with [h] lies upstream of the grammar. Linguistic and non-linguistic stimuli were created using sound samples recorded as speech: the linguistic items were full syllables, while the non-linguistic items were fricative noise bursts. These stimuli were then used to examine learners’ perceptual abilities with respect to [h]: specifically, we elicited the mismatch negativity (MMN) to assess detection of [h].

### Mismatch Negativity

The MMN is a response manifested by a negative-going component occurring approximately 200 ms after stimulus presentation that indicates automatic (pre-attentive) detection of physical deviance in a stream of acoustic stimuli, usually elicited in an oddball paradigm (Näätänen, 1999; see also Phillips et al., 2000; Eulitz and Lahiri, 2004). There is evidence that the MMN is modulated, or exclusively elicited, by changes which cross phonological category boundaries: Phillips et al. (2000) found that voice onset time (VOT) differences that resulted in stimuli being categorized as separate instances of a single phoneme did not elicit the magnetic equivalent of the MMN (the mismatch field); VOT differences that resulted in stimuli being categorized as instances of two distinct phonemes did elicit the response (cf. Sharma and Dorman, 1999).

If French speakers’ difficulties with [h] are due to its acoustic non-salience, then no MMN should be obtained in either the linguistic or non-linguistic condition. If, however, their difficulties reflect a problem in building a phonological representation for [h], then they should be able to perceive this segment when it is processed non-linguistically (as in Werker and Tees, 1984), but not when it is processed linguistically. This would be revealed by an asymmetry in the elicitation of the MMN: we would expect to find an MMN response in the non-linguistic condition paired with a lack of MMN response in the linguistic condition.

### N100, P3a, Late Negativities

In addition to MMN responses, ERP studies on sound discrimination often report modulations of other components that we might observe in our data. The N100 is an early negativity around 100 ms that precedes the MMN and is thought to primarily reflect very early cortical processes regarding the physical and temporal characteristics of an auditory stimulus, largely independent of whether it serves as a standard (frequent) or deviant (infrequent; Näätänen, 1999). However, sometimes its amplitude increases for deviants, which may be related to reduced habituation (Sokolov et al., 2002). In a study on vowel discrimination, Molnar et al. (2014) found a significantly greater N100 amplitude for a given stimulus type in the deviant condition as compared to the standard condition. Another ERP component often observed following an MMN is the P300, with its subcomponents P3a and P3b. The fronto-central P3a (around 250 ms) is part of an orientation response toward unexpected deviants, whereas the later, parietally distributed P3b (around 350 ms) reflects the updating of environmental representations in working memory based on the conscious categorization of a stimulus as a deviant. P300s are typical for attended oddballs, where the task is to count or respond to auditory deviants (Näätänen et al., 2007). In unattended oddballs, where participants are instructed to ignore the auditory stimuli and focus on MMN-irrelevant visual stimuli (e.g., silent videos), the P3a is taken to index the involuntary shift of attention toward a salient stimulus in the (otherwise unattended) auditory stream (Näätänen, 1999, Näätänen et al., 2007). Lastly, a “late mismatch negativity” or “late discriminative negativity” has been reported between 400 and 700 ms post-deviant onset, especially for word-like stimuli, and particularly in children (Cheour et al., 2001) and young adults (Mueller et al., 2008). Its functional significance is not well-understood, but its latency points to controlled processes, as compared to the ‘automatic’ pre-attentive MMN.

## Materials and Methods

### Stimuli

For the linguistic condition, the syllables [ᴧm] ‘um,’ [hᴧm] ‘hum,’ and [𝜃ᴧm] ‘thumb’ were used. The vowel [ᴧ] was selected to minimize coarticulation effects on [h]: given that [h] manifests acoustically as a voiceless vowel, [ᴧ] was selected as its articulation most closely approximates a positionally neutral vocal tract. [𝜃] was selected as a distracter since, as discussed above, it is a low-intensity fricative and is also absent from French. Three instances of each item were recorded by a female native speaker of English, each with falling intonation; all tokens were used in an adapted oddball paradigm, described below (Phillips et al., 2000).

**Table 1** (upper) provides each consonant’s duration, along with the total duration of each token in the linguistic condition, measured in Praat (Boersma and Weenink, 2010). As can be seen, the duration values vary, as the samples reflect natural speech. Given that the task was designed to assess participants’ detection of [h], the samples were not edited to alter overall duration, as any manipulation that would bring the overall duration of the [ᴧm] and [hᴧm] items closer together necessarily involves lengthening the vowel or nasal in [ᴧm] or shortening the vowel or nasal in [hᴧm]. This would have made the interpretation of any MMN component unclear as it introduces an additional salient cue that could allow participants to distinguish among stimulus types: rhyme length. It would have been impossible to determine if participants were detecting the presence or absence of [h] or were instead responding to differences in rhyme length.^2^

**Table 1:** List of Stimuli used in the present MMN study.

Condition	Token		Initial consonant duration (ms)	Vowel duration (ms)	Total duration (ms)
Linguistic	*um1*	[ᴧm]	0	188	416
	*um2*	[ᴧm]	0	203	439
	*um3*	[ᴧm]	0	187	441
	*hum1*	[hᴧm]	106	162	479
	*hum2*	[hᴧm]	133	185	516
	*hum3*	[hᴧm]	92	181	497
	*thumb1*	[𝜃ᴧm]	149	201	554
	*thumb2*	[𝜃ᴧm]	218	222	636
	*thumb3*	[𝜃ᴧm]	179	192	580
Non-linguistic	*f1*	[f]	133		133
	*f2*	[f]	198		198
	*hf1*	[hf]	104		306
	*hf2*	[hf]	132		336
	*thf1*	[𝜃f]	215		417
	*thf2*	[𝜃f]	140		330

For the non-linguistic condition, in order to create a series of stimuli that paralleled those in the linguistic condition, an additional distracter consonant was needed to serve as the consistent ‘base’ of the items, much as [ᴧm] was the ‘base’ of the linguistic items. [f] was selected for this as it is another low-intensity fricative; it is also present in both English and French. The ‘linguistic’ recordings of [hᴧm], [𝜃ᴧm], and an additional syllable [fᴧn], were manipulated to create fricative noise bursts corresponding to [f], [hf], and [𝜃f]. Two tokens of each noise burst sequence were created, and both tokens were used in the task. As in the linguistic condition, the non-linguistic items were not manipulated to adjust for differences in overall length in order to avoid introducing length of [f] as a potential cue for discrimination. **Table 1** (lower) provides the duration of each initial consonant extracted from the recordings of [fᴧn], [hᴧm], and [𝜃ᴧm], as well as the total duration of each non-linguistic condition stimulus.

In order to prevent participants from using a perceived delay in stimulus presentation (due to non-perception of [h]) as a reliable identifying cue for [h]-initial items in both linguistic and non-linguistic conditions, stimulus onset asynchrony (SOA) ranged from 750 to 850 ms (average 800 ms). With a variable SOA, participants would not be able to rely on a longer SOA to identify [h] items, and a shorter SOA to identify non-[h] items. This facilitates the interpretation of the ERP data: any MMN that is elicited reflects detection of deviant items based on the items themselves, and not on the timing of the presentation of deviant items.

### Participants

Seventeen native French speakers participated in this study; seven were recruited in Montreal, QC, Canada and 10 in Calgary, AB, Canada. Participants from Montreal were enrolled in English classes at Université du Québec à Montréal; two were French (on exchange from France); the remaining five were Canadian. All Montreal participants were recruited from courses designed for students whose proficiency is advanced beginner to low intermediate, as demonstrated by performance on a placement test or through satisfactory completion of courses designed for students of lower proficiency. Participants from Calgary had been living in Calgary minimally for 2 years. Three were originally from Quebec, five were from France, one was from Switzerland, and one was from Morocco.^3^ Of the Calgary French speakers, six reported using English most of the time in their daily lives at home and work (upward of 75% of the time), one reported equal usage of French and English, and two reported using English less than 30% in their daily lives at home and work. Self-assessed ratings of proficiency ranged from high intermediate to native-like, and all participants were greater than 80% accurate on a short written cloze test (the Michigan ECPE Grammar Test). Data from the two groups are collapsed below, as additional analyses of the ERP data including factor ‘Testing Site’ (Montreal vs. Calgary) indicated that neither our main results nor their interpretation were influenced by this factor.^4^

Twenty-four native English speakers were recruited as controls: nine students from McGill University in Montreal, and 15 students from the University of Calgary. All participants were right-handed and provided written informed consent, as approved (along with the protocol) by the Research Ethics Boards at both universities.

### Procedure

All participants were fitted with an electrode cap (Ag-AgCl electrodes) that recorded activity from 11 scalp electrodes (Fz, Cz, Pz, FP1, FP2, F3, F4, C3, C4, F7, F8) with a forehead ground and earlobes reference. EOG channels (both horizontal and vertical) were also recorded to monitor eye movement. Auditory stimuli, which participants were instructed to ignore while watching a silent video (unattended oddball), were presented by insert earphones to both ears. Participants in Montreal were seated in an electrically shielded sound attenuated booth; participants in Calgary were seated in a quiet room.

Stimuli were presented in an adapted oddball paradigm (Phillips et al., 2000): at the acoustic level, since multiple tokens of each test syllable were used, no single token occurs with sufficient frequency to be considered a standard. At an abstract level, however, a clear pattern of standard and deviant items emerges. This paradigm was chosen to ensure that any effect observed reflects consultation of stored memory traces. Four blocks of stimuli were presented: (1) a linguistic condition block with [hᴧm] items as standards (80%) and [ᴧm] and [𝜃ᴧm] items as deviants (10% each), (2) a linguistic condition block with [ᴧm] items as standards (80%) and [hᴧm] and [𝜃ᴧm] items as deviants (10% each), (3) a non-linguistic condition block with [hf] items as standards (80%) and [f] and [𝜃f] items as deviants (10% each), and (4) a non-linguistic condition block with [f] items as standards (80%) and [hf] and [𝜃f] items as deviants (10% each). All participants were presented with alternating blocks of linguistic and non-linguistic stimuli, and the order of presentation of blocks was counterbalanced to create four versions of the experiment; this was done to avoid sequence effects, such as the confound of fatigue, in any given condition.

EEG data were recorded continuously using Neuroscan amplifiers with a sampling rate of 500 Hz. All data were analyzed offline using EEProbe [Advanced Neuro Technology (ANT), the Netherlands]: the data were subject to offline bandpass filtering (0.5–30 Hz), and averages for each test condition were computed separately. The data were subjected to eyeblink and movement artefact rejection, after which each individual data set contributed 675 trials to each of the four ‘standard’ conditions and 84 trials to each of the four ‘deviant’ conditions on average.^5^ ERP averages were time-locked to the onset of the stimulus item; epochs (-70–800 ms) included a 70 ms prestimulus baseline.

**FIGURE 1:**
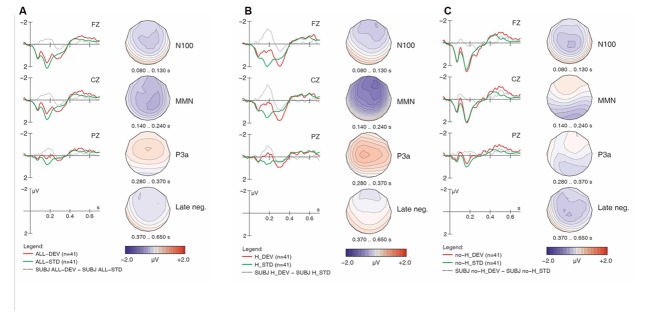
**Overall ERP mismatch effects: collapsed across all sub-conditions and both groups (A); collapsed across both groups for [h] items (B); collapsed across both groups for non-[h] items (C).** Negativity is plotted upward; vertical axis at 0 ms indicates stimulus onset. Voltage maps depict the distribution of the difference wave in the time windows used for statistical analysis.

**FIGURE 2:**
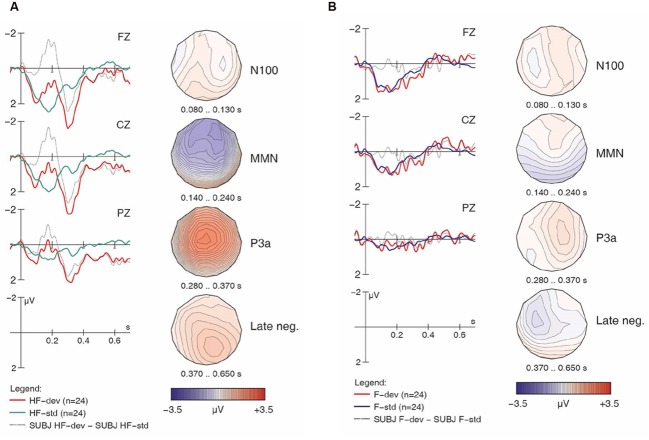
**Native English responses to [hf] (A) and [f] (B) items, contrasting standards and deviants, with difference wave (deviant minus standard).** Negativity is plotted upward.

**FIGURE 3:**
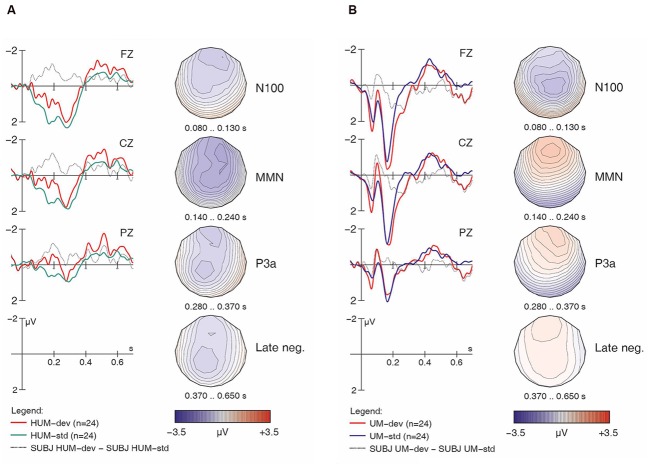
**Native English responses to [hᴧm] (A) and [ᴧm] (B) items, both standards and deviants, with difference wave.** Negativity is plotted upward; vertical axis at 0 ms indicates stimulus onset.

**FIGURE 4:**
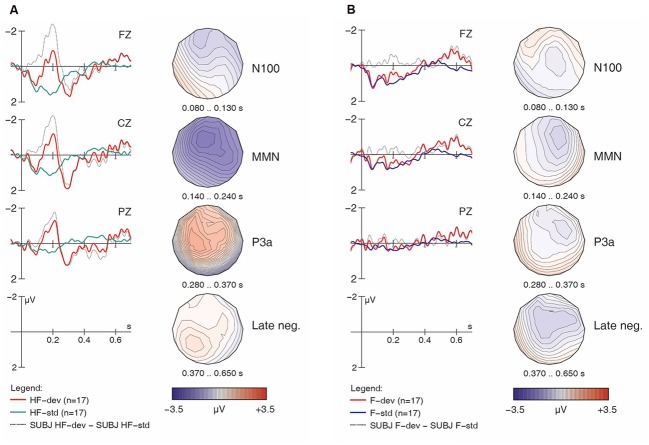
**Learner responses to [hf] (A) and [f] (B) items, both standards and deviants, with difference wave and voltage maps.** Negativity is plotted upward; vertical axis at 0 ms indicates stimulus onset.

**FIGURE 5:**
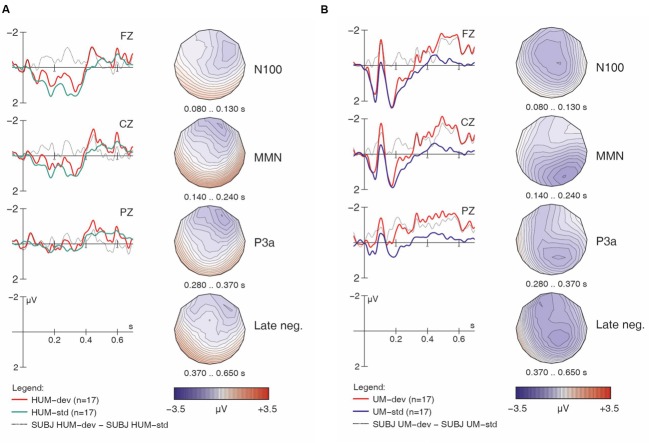
**Learner responses to [hᴧm] (A) and [ᴧm] (B) items, both standards and deviants, with difference wave.** Negativity is plotted upward; vertical axis at 0 ms indicates stimulus onset.

Based on previous research and visual inspection of the grand average waveforms, four time intervals were selected to quantify ERP components: an 80–130 ms time window for analysis of the N100, 140–240 ms for the MMN, 280–370 ms for the P3a, and 370–650 ms for late components. Mean amplitudes for each time window were analyzed using a global ANOVA with the between-subject factor *Group* (English, French) and the within-subject factors *H-presence* (with [h], without [h]), *Type* (linguistic, non-linguistic), *Match* (standard, deviant), and *Electrode* (Fz, Cz, Pz).^6^ To avoid any confounds between mismatch effects and physical differences among stimuli, in all analyses we always compare a given stimulus (presented as a deviant) against itself (presented as a standard), as illustrated in **Figures 1**–**5**. Analyses at lateral electrodes were also performed; however, since all relevant effects were already reflected at midline electrodes (see figures), we decided not to include the lateral analyses. In line with recommendations in the literature (e.g., Nieuwenhuis et al., 2011), our statistical analyses follow a strict hierarchical order. First, starting with a global ANOVA, we will report only relevant main effects and interactions involving the factor *Match*. Second, only those effects that reached significance (*p* < 0.05) in the global ANOVA will be followed up by step-down analyses (within groups, subsets of conditions, or at individual electrodes) to clarify the underlying data pattern.

## Results

For each of the ERP patterns, we provide an overview of observations from visual examination of the waveforms accompanied by corresponding statistical analyses. **Figure 1A** displays the overall ERP mismatch effects (deviants vs. standards) collapsed across all sub-conditions and both groups. Note that comparisons are always made between identical physical stimuli in the standard vs. deviant conditions in order to rule out any confound of the mismatch effects with ERP components due to physical differences between stimuli. We can see that the two waveforms show a typical pattern of onset components (P100, N100, P200), which are followed by a relative negativity between 400 and 650 ms. While the two conditions are virtually identical during the first 80 ms, they then start diverging. Deviants elicited an enhanced fronto-central negativity in the N100 time range, followed by a broadly distributed MMN between 140 and 240 ms. Between 250 and 370 ms, deviants then display a fronto-central positive waveform (P3a), followed by a small and broadly distributed late sustained negativity that lasts almost until the end of the average window (650 ms). The four voltage maps illustrate the scalp distribution for each of these effects (deviant minus standard) in representative time windows that also underlie our statistical analyses.

As will be seen, the four mismatch effects described above (N100, MMN, P3a, late negativity) are not evenly distributed across the four sub-conditions or two language groups. One difference between sub-conditions is illustrated in **Figures 1B,C**. Stimuli containing [h] (collapsed across [hf] and [hᴧm] in **Figure 1B**) seem to elicit a clear MMN around 200 ms and a subsequent positivity (P3a), whereas stimuli lacking [h] (collapsed across [f] and [ᴧm] in **Figure 1C**) display much smaller ERP differences, primarily in very early and late time windows (around 100 and after 400 ms, respectively). We begin our examination with the MMN.

### MMN Components

A global repeated measures ANOVA for the 140–240 ms time window revealed the significant effects in **Table 2**.

**Table 2:** Global ANOVA (MMN) for Group comparisons and within group follow-ups.

Effect source		Group contrast (*n* = 41)		Controls (*n* = 24)		Learners (*n* = 17)	
	dF	*F*-value	*p*-value	*F*-value	*p*-value	*F*-value	*p*-value
*Match*	1	**9.56**	**0.0037**	2.67	0.1158	7.06	0.0172
*Match x H-presence*	1	**10.19**	**0.0028**	10.01	0.0043	2.21	0.1563
*Match x H-presence x Electrode*	2	**16.3**	**<0.0001**	12.8	0.0002	5.12	0.0208
*Match x Type x Electrode*	2	**9.34**	**0.0013**	5.73	0.0162	4.28	0.0334
*Match x H-presence x Type x Group*	1	**4.43**	**0.0419**				
*Match x H-presence x Type*	1			0.37	0.5478	**13.86**	**0.0019**

*Bold values indicate the relevant findings*.

The *Match* main effect reflects the MMN components for deviants (compared to standards) across groups and conditions. However, this main effect is qualified by an even stronger *Match x H-presence* interaction, corresponding to the presence of the MMN with [h] items (**Figure 1B**) and its absence with non-[h] items (**Figure 1C**). Moreover, the highly significant three-way interaction of *Match x H-presence x Electrode* indicates that the MMN in [h] items is most prominent at frontal electrodes (cf. **Figure 1B**) and not evenly distributed along the anterior–posterior axis of the midline. Similarly, the *Match x Type x Electrode* interaction points to different MMN distributions in linguistic vs. non-linguistic conditions. Note that all of these effects were shared between the controls and learners with no evidence of group differences (all *p*-values > 0.2 for respective interactions with factor *Group*).

Importantly, the analysis finds a four-way interaction of *Match x H-presence x Type x Group*, suggesting group differences in the responses, and prompting follow-up analysis and separate examinations of the ERP waveforms for the controls and the learners. We will examine the controls first, as their results serve as a baseline for interpreting the results obtained from the learners. For the non-linguistic condition, **Figure 2A** shows the control group’s responses to [hf] items, comparing [hf] as standards and [hf] as deviants; **Figure 2B** shows this group’s responses to [f] items.

Visual inspection of **Figure 2A** suggests that [hf] as a deviant elicited a large and broadly distributed MMN (with a fronto-central maximum), which would indicate that the presence of [h] in [hf] was automatically detected by these participants. It is followed by a large P3a-like positivity. In **Figure 2B**, however, the waveforms provide no indication that an MMN was elicited. This is surprising given the phonemic status of [h] in English, as it suggests that these native English speakers were unable to detect deviant [f] tokens among standard [hf] tokens; however, this finding is in line with the significant interaction of *Match x H-presence* in the global ANOVA.

Turning to the linguistic condition, **Figures 3A,B** show the English group’s responses to [hᴧm] and [ᴧm] items, respectively. Similar to the non-linguistic contrasts, an MMN is only visible for [h] items (i.e., in the [hᴧm] condition), not for the non-[h] items. This pattern largely mirrors what we already saw in **Figures 1B,C**. As seen in **Table 2**, the within-group follow-up ANOVA found no significant interaction of *Match x H-presence x Type* for the English controls, indicating that the observed MMN effects are significant in both the non-linguistic and linguistic [h] conditions.

Having established the patterns for native English speakers, we now turn to the learners’ results, beginning with the non-linguistic condition. If French speakers’ difficulties stem from the acoustic properties of [h], we expect across-the-board group differences, including for the non-linguistic stimuli. However, as with the English controls, the learners’ ERP grand averages suggest that [hf] as a deviant elicited a large MMN and a subsequent P3a (**Figure 4A**), while [f] as a deviant did not (**Figure 4B**). This does not point to any differences in ERP patterns between the learners and controls for the non-linguistic condition.

Turning now to the linguistic condition, **Figures 5A,B** show the learners’ responses to [hᴧm] and [ᴧm] items, respectively. While the English controls’ results suggested a clear MMN for [hᴧm] items, the learners’ data is less clear. In fact, while deviant [hᴧm] stimuli seem to have elicited larger negativities both in the N100 time range and in later time windows (roughly 250–550 ms), hardly any difference between conditions is seen in our standard MMN interval (140–240 ms), where the controls had displayed their main response. Moreover, the later negativities have a frontal distribution. Overall, this pattern appears atypical and suggests that the learners did not detect the presence of [h] in [hᴧm] among [ᴧm] standards relying on the same pre-attentive processing mechanisms typical for native English speakers. Indeed, the within-group follow-up analysis revealed a significant interaction of *Match x H-presence x Type* (*F*_1,16_ = 13.86, *p* < 0.002) for the MMN in the learner group.

For the [ᴧm] items, as with the English controls, visual inspection of the waveforms in **Figure 5B** does not suggest an MMN component; instead, we find an enhanced (more negative) N100 in the deviant condition. Additionally, a pattern of sustained negativity (~200–700 ms) reminiscent of that found with [hᴧm] items (in **Figure 5A**) is also seen here, however, with a more posterior distribution.^7^

Importantly, the MMN was observed in the controls for both [hf] and [hᴧm] items, but in the learners, it was *only* observed for [hf] items: while the learners showed a highly significant *H-presence x Type x Match* interaction (*p* < 0.002), this effect was completely absent in the controls (*F* < 1). Conversely, in the controls, the strongest effects were the *H-presence x Match* (*p* < 0.005) and *H-presence x Match x Electrode* interactions (*p* < 0.0002), reflecting the presence of MMNs for [h] items and their absence in non-[h] items (*across* linguistic and non-linguistic stimuli).

As the most important interactions qualifying the *Match* main effect in the global ANOVA involved *H-presence*, the next follow-up analysis investigated MMN effects separately for [h] items (**Figures 2A**, **3A**, **4A**, and **5A**) and for non-[h] items (**Figures 2B**, **3B**, **4B**, and **5B**). These results are summarised in **Table 3**.

**Table 3:** ANOVAs (MMN) separately for [h]-presence and [h]-absence.

Effect	Group contrast with [h]		Controls with [h]		Learners with [h]		Group contrast no [h]	
Source	*F*-value	*p*-value	*F*-value	*p*-value	*F*-value	*p*-value	*F*-value	*p*-value
*Match*	**15.18**	**0.0004**	7.8	0.0103	8.7	0.0094	0.31	0.5803
*Match x Electrode*	**6.92**	**0.0062**	2.57	0.1068	4.33	0.0444	8.13	0.0018
*Match x Type x Group*	**4.43**	**0.0417**	-	-	-	-		
*Match x Type*			0.01	0.9266	**8.61**	**0.0097**		
*Match x Type x Electrode*							9.39	0.0014

*Bold values indicate the relevant findings*.

**Table 3** shows that, for responses to [h] items, a highly significant main effect of *Match* and a *Match x Electrode* interaction were found across both the controls and learners, pointing to reliable fronto-central MMN components that were most prominent at Fz (*p* < 0.0001) and Cz (*p* < 0.001), but still significant at Pz (*p* < 0.005). Importantly, we also found a significant *Match x Type x Group* interaction that, again, pointed to the group differences mentioned above. Follow-up analyses within each group (middle rows in **Table 3**) revealed that only the learners had a significant *Match x Type* interaction (*p* < 0.01), whereas this effect was absent in the controls (*F* < 1). The learner data were thus subject to further follow-up analyses for each of the two [h] sub-conditions. These ANOVAs revealed a highly significant main effect of *Match* for [hf] (*F*_1,16_ = 11.22, *p* < 0.005), but no effect for [hᴧm] items (*F* < 1). This confirms our interpretation of the ERP waveforms: for the controls, a significant MMN is obtained for *both* [hf] (*p* < 0.04) and [hᴧm] (*p* < 0.03), whereas the learners elicited an MMN for deviant [hf] (*p* < 0.005), but not for deviant [hᴧm] (*F* < 1).

In the group contrast of no-[h] items (rightmost columns in **Table 3**), no *Match* main effect was found (*F* < 1), whereas a *Match x Electrode* interaction reached significance, which was qualified by a 3-way interaction of *Match x Type x Electrode*. The corresponding follow-up analyses within each sub-condition revealed a significant interaction between *Match* and *Electrode* for [ᴧm] (*F*_2,78_ = 14.78, *p* < 0.0001), but not for [f] (*F* < 1), confirming the absence of an MMN in the latter condition in both groups. Importantly, the highly significant *Match x Electrode* interaction for [ᴧm] items does not point to an MMN either. Instead, it reflects both a relative frontal positivity (especially in the controls; **Figure 3B**) and early parts of a relative posterior negativity (especially in the learners; **Figure 5B**). Separate follow-up analyses of [ᴧm] data from all participants at each electrode revealed a significant main effect of *Match* only at Pz (*F*_1,39_ = 5.67, *p* = 0.03); this reflects the observed posterior negativity for deviant [ᴧm] items across groups.^8^

In the following sections, we briefly address statistical analyses for ERP components observed in other time intervals.

### P3a Component

Recall that in addition to the MMN discussed above, **Figures 2A** and **4A** also reveal a large fronto-central P3a component near the midline (i.e., at Fz and Cz). As the participants for this study were instructed to ignore the acoustic stimuli and simply watch the silent movie, this P3a suggests that these deviant stimuli were particularly salient, triggering an involuntary shift of attention (Näätänen, 1999). By contrast, no such component was observed with non-[h] items (**Figures 3A** and **5A**). A global repeated measures ANOVA for the 280–370 ms post-stimulus time window yielded the relevant effects in **Table 4**.

**Table 4:** Global ANOVA (P3a).

Effect source	Group contrast	
	*F*-value	*p*-value
*H-presence x Match*	5.48	0.0245
*H-presence x Type x Match*	10.76	0.0022
*H-presence x Type x Match x Electrode*	5.3	0.0172
*Type x Match*	23.15	<0.0001

The interactions involving *H-presence* prompted a follow-up repeated measures ANOVA with the data divided based on presence or absence of [h] in the test items. Where analysis of the [h]-less items yielded no effects or interactions, analysis of the [h] items found a significant main effect of *Match* (*F*_1,39_ = 4.86, *p* = 0.0334) and a significant interaction of *Type x Match* (*F*_1,39_ = 28.03, *p* < 0.0001).

An additional follow-up analysis was run for the [h] items, dividing the data based on type. A repeated measures ANOVA found a significant main effect of *Match* for the non-linguistic [h] items (*F*_1,39_ = 22.96, *p* < 0.0001); however, no effects were found for the linguistic [h] items (*F*_1,39_ = 2.98, *p* = 0.0932). This result confirms that deviant [hf] elicited a significant P3a component for both the controls and learners, but [hᴧm] did not.

### N100 Components

For the 80–130 ms post-stimulus time window (N100), a global repeated measures ANOVA revealed a significant interaction between *Type* and *Match* (*F*_(1,39)_ = 5.01, *p* = 0.031). A follow-up repeated measures ANOVA found a main effect of *Match* (*F*_1,39_ = 7.92, *p* = 0.0076) for linguistic condition items, but not for non-linguistic items (*F* < 1). These results indicate the presence of an enhanced (more negative) N100 in response to deviant items in the linguistic condition, but not in the non-linguistic condition.

### Late Negativity

Between 370 and 650 ms post-stimulus onset, a broadly distributed negative deflection is visible in linguistic as compared to non-linguistic conditions (*Type* main effect: *F_1,39_* = 30.53, *p* < 0.0001). The negativity is particularly pronounced in linguistic mismatch conditions (*Type x Match*: *F_1,39_* = 10.70, *p* = 0.0022). An additional *Match x Group* interaction (*F_1,39_* = 6.03, *p* = 0.018) points to a larger late mismatch effect in the learners (*F_1,16_* = 8.03, *p* = 0.012) than in the controls (*F_1,23_* < 1).

### Results Summary

In our analysis of overall ERP patterns, we found that only deviant stimuli containing [h] elicited any reliable MMN effects. Further analysis of group differences revealed that in the non-linguistic condition, English control and learner responses were similar, in that both responded with significant MMNs. In the linguistic condition, however, responses differed, in that only the controls responded with a significant MMN. Where [hᴧm] items served as deviants among [ᴧm] standards, the controls showed a significant MMN, suggesting that they were able to detect the presence of [h] on deviant items, while the learners did not show a clear MMN component, which in turn suggests that they were unable to automatically detect the presence of [h] on deviant items; however, the deviant condition response had a general increase in negativity, particularly during later time windows. Furthermore, an ANOVA revealed a significant interaction between *Match* and *Type* in the learners’ responses only. Where [ᴧm] items served as deviants among [hᴧm] standards, neither the controls nor the learners showed a significant MMN component in their responses; however, both groups did show an N100 effect, and the learners also showed greater overall negativity in their responses, again, particularly in later time windows.

## Discussion

This study examined the abilities of native English speakers and learners in detecting the presence and absence of English [h] as both linguistic (full syllable) and non-linguistic (noise burst) items. Our results found differences in the pre-attentive processing of [h] and [h]-less items, as well as group differences in the pre-attentive processing of [h] as a linguistic (but not non-linguistic) item between English and French speakers. We discuss each of these findings below.

### No MMN for Non-[h] Items

Considering first the responses obtained for non-[h] items, interpreting these is not straightforward: neither language group showed an MMN in either the linguistic or non-linguistic condition. This result is surprising for the controls, as we would expect them to have good discriminatory abilities for [h] owing to its phonemic status in English. It is also surprising for the learners in the non-linguistic condition as they were shown to behave like native English speakers on the [h] stimulus items. Essentially, these results suggest that the unexpected *presence* of [h] was salient, but its unexpected *absence* was not; that is, the difference between deviant and standard items was salient when the deviant introduced new acoustic material, but the difference was not salient when the deviant was a subset of the information contained within the standard.

A potential explanation for this finding stems from the interpretation of similar findings in the speech recognition literature. In the Featurally Underspecified Lexicon model of Lahiri and Reetz (2002), speech recognition is achieved through evaluation of perceived features against those stored for the segments that make up candidate morphemes using a ternary logic system of *match*, *no-mismatch*, and *mismatch*. If the input speech stream presents features that are different from those of the segments of a given candidate, the result is judged as a *mismatch* and the candidate is discarded. If the features of the input match those of a given candidate, the result is judged as a *match* and the candidate is assigned a high score, resulting in selection of that candidate. If, however, the input presents features that do not match a given candidate but at the same time do not conflict with those of the candidate, namely, in the case of features that are underspecified in stored representations, then the result is judged as *no-mismatch*, which allows the candidate to remain an option for selection in recognition. The expected asymmetry between *mismatch* and *no-mismatch* for place of articulation has been shown to be reflected in ERP components by Eulitz and Lahiri (2004). These authors found that in cases where the deviant stimulus items presented a feature that was a *mismatch* when compared with those features stored for the standard, the MMN component elicited had a greater amplitude and earlier peak latency than that elicited by deviant items that were a *no-mismatch*. A similar asymmetry was observed by Schluter et al. (2016) in the perception of fricative noise bursts for [s] and [h]: a *mismatch* deviant stimulus type elicited a larger MMN than a *no-mismatch* deviant, supporting the view that the phonological representation of [h] has no place features (see Phonological Consequences of the Phonetic Properties of [h]). In the case reported here, all the features present in our deviant [h]-less items are a subset of the features present in our standard items, which are stored for evaluation of deviance. The deviant [h]-less items in these conditions thus do not mismatch the stored representation for the standard [h]-items, as the [h]-less deviant does not present any features that are absent from the stored representation.

Given that the asymmetric finding in our data (MMN present for [h] items but absent for non-[h] items) was unexpected, our proposed account is necessarily preliminary and somewhat speculative. However, the consistency of this pattern across both linguistic and non-linguistic materials in native speakers (and at least for non-linguistic stimuli in the learners) suggests that these asymmetries merit further investigation in future work.

### No MMN for Linguistic [h] in French Speakers

For our main research question, the pattern of group differences was of greatest interest. Regarding the [h] stimulus items, our results lead us to reject the hypothesis that the difficulties that learners have with English [h] are due to this segment’s acoustic properties: in the non-linguistic condition, they performed like native English speakers, in that a deviant [hf] item elicited both a large significant MMN as well as a large significant P3a. This ERP pattern suggests that both groups reliably detected the mismatch in an automatic (pre-attentive) fashion (MMN) and that the mismatch was salient enough to trigger an orienting response (P3a), indicating listeners’ shift of attention toward the eliciting event (Näätänen, 1999). In the linguistic condition, however, only the controls showed a clear and significant MMN response (without a P3a).

Our results in the linguistic condition are consistent with LaCharité and Prévost’s (1999) perception study, which involved real word stimuli. Both studies suggest that learners are unable to perceive [h] in the speech stream. The fact that the French speakers in our study were able to perceive its presence in the non-linguistic condition provides strong evidence against the hypothesis that the acoustic non-salience of [h] is the root of the problem. Recall that in the linguistic condition, three different tokens of each stimulus item were presented throughout the task such that there was no single consistently produced standard item. This ensured that discrimination could not be made on the basis of fine acoustic detail. Instead, the methodology employed forced participants to make use of an abstract mental representation to characterize the standard in memory traces. In the linguistic condition, these representations would be phonological in nature, and these same speakers’ inability to detect the presence of [h] in the linguistic condition thus strongly suggests that the difficulty lies in constructing and accessing a phonological representation for [h].

This interpretation, however, may appear to be challenged by recent ERP results reported in White et al. (2015): in their study, learners performed like native English speakers in an attended auditory discrimination task (attended oddball) using stimuli that are similar to those used in the linguistic condition in our study; however, their results revealed significant MMN and P300 components for both groups. Upon further examination, this apparent contradiction is likely due to differences in task and stimulus choice between the two studies, as discussed below.

#### Task Effects and Attention

In White et al.’s (2015) study, participants were given an explicit auditory discrimination task that required them to pay close attention to the stimuli being presented (attended oddball), whereas our participants were instructed to ignore the auditory stimuli, and watched a silent movie instead (unattended oddball). It is well-known that only attended (but not unattended) oddballs require listeners to consciously categorize stimuli as standards and deviants (Näätänen, 1999, Näätänen et al., 2007), thus accounting for the consistent finding of posterior P300 (P3b) components following the MMN effect in White et al.’s (2015) study, but not in ours. In contrast, the pre-attentive MMN component itself is usually not affected by such task requirements. Under certain circumstances, however, the difference between paying attention to a phonetic contrast or not may substantially change the cognitive processing, including those operations reflected by MMN effects in L2 learners, as pointed out by White et al. (2015). White et al. (2015) discussed this data pattern as evidence supporting certain assumptions of the automatic selective perception (ASP) model of Strange (2011). According to the ASP, native contrasts in the L1 are processed automatically by recruiting highly over-learned ‘selective perception routines’ that reliably discriminate between L1 phoneme categories. For L2 learners, these routines are not available for contrasts that do not exist in their L1, resulting in less automatic processing and requiring more attentional resources. Thus, the ASP model predicts that discrimination of non-native contrasts may be more successful in tasks that require L2 learners to pay special attention to the contrast, as would be the case in attended oddballs, but not in unattended oddballs. This line of argumentation would, therefore, provide an account for the difference between White et al.’s (2015) results and those reported in our study.

One might wonder, however, how a shift of attention may affect the MMN, given that it is generally viewed as an ERP component reflecting very early *pre-attentive* processing (Näätänen, 1999, Näätänen et al., 2007). We believe that, if attention shifts do indeed increase discriminability as reflected by the MMN, the underlying mechanism of this component must rely on a better representation of the contextual memory trace of the *standard stimuli* (against which the deviant is compared), instead of different processing of the deviant; similar ideas were previously put forward by Sussman (2007). In the case of [h], this mechanism would arguably predict a stronger benefit of attended (vs. unattended) oddballs for conditions where the [h]-items served as standards. This was the case for our [f] and [ᴧm] deviants, the ERP data for which were *collected* against standards [hf] and [hᴧm], respectively, although we *analyzed* them in comparison to physically identical standard stimuli (i.e., [f] and [ᴧm] presented as standards). Recall that these two conditions of our unattended oddball did not elicit any ERP effects in either group (cf. **Figures 2B** and **4B**). Interestingly, as predicted above, White et al. (2015) did find clear mismatch effects (MMNs and P300s) for *all* of their [h] contrasts and, surprisingly, behavioral performance in their discrimination task (attended oddball) was even somewhat better when the [h] item served as a standard rather than a deviant. While this complex and apparently inconsistent pattern of findings would otherwise be difficult to explain, it follows straightforwardly from the ASP model (Strange, 2011) and the proposed underlying mechanism affecting the MMN (Sussman, 2007; White et al., 2015), lending strong support to both proposals.

To summarize, certain non-native contrasts are difficult to process, especially in “testing conditions that are similar to natural language processing” (Werker and Tees, 1984). Neurobiological evidence shows that French speaking English learners are not able to distinguish between *apple* and *happle* (White et al., 2015) nor do they automatically discriminate between word-like stimuli such as [ᴧm] and [hᴧm] (in the present study). On the other hand, embedding the [h] contrast in non-linguistic stimuli ([f] vs. [hf]) and drawing their attention to the [h] contrast (attended oddball) help to improve the discriminability of minimal pairs, even at a pre-attentive level.

Together, the data from White et al. (2015) and the present study suggest that the difficulties observed with these learners may result from the lack of an appropriate phonological representation for [h] rather than a general inability to hear the [h]-Ø contrast. This interpretation is further supported by another finding in White et al.’s (2015) study: for learners at lower levels of proficiency, words and pseudowords (*happy* and *appy*) both elicited *small* N400 amplitudes that are characteristic of real words but not pseudowords in native speakers and high-proficiency L2 learners.^9^ In fact, these small N400s were very similar to those found for words (*foolish, apple*) but not pseudowords (*oolish, fapple*) in the same participants for a native and easy-to-process [f]-Ø contrast. This suggests that, no matter whether they hear *apple* or *happle*, learners at relatively low levels of proficiency access the lexical entry for *apple* equally easily (White et al., 2015). Moreover, unpublished N400 data from our lab indicate that minimal pairs (*eat* vs. *heat*) are processed like homophones (e.g., *thyme* and *time*) by learners, such that *curly air* sounds acceptable and does not elicit an N400 effect, whereas it does in native speakers (Mah et al., in preparation, unpublished).^10^

#### Choice of Stimuli

While different tasks and the role of attention are likely to have contributed to differences between our findings and those in White et al. (2015), other factors must be considered as well. Most importantly, White et al. (2015) selected a different vowel for their stimuli: our stimuli were constructed using [ᴧ], a mid central vowel, whereas White et al. (2015) selected *a*, a low vowel.^11^ Although [h] phonologically lacks place features (see Phonological Consequences of the Phonetic Properties of [h]), one consequence of this is that the phonetic quality of [h] is highly influenced by context; specifically, [h] is produced using the vocal tract shape of an adjacent vowel (Keating, 1988). The choice of [ᴧ] for our stimuli was deliberate, as [ᴧ] most closely approximates a positionally neutral vocal tract, resulting in the least amount of audible overlap between this segment and the preceding [h]. A low vowel, by contrast, has the effect of narrowing the vocal tract in the vicinity of the pharynx, with the overall acoustic effect being an [h] which is much like that of a voiceless uvular continuant. Notably, devoiced uvular rhotic continuants ([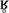]) are present in French (Walker, 2001); White et al.’s (2015) results may thus reflect French speakers’ sensitivity to devoiced rhotics rather than their sensitivity to [h]. Both White et al.’s (2015) and our own N400 data, which together examine responses to [h]-initial words in a variety of vowel contexts, suggest that French speakers display problems in perception similar to those in production, and rely on non-native-like representations.

### N100 and Late Negativity

Similar to previous oddball studies on phoneme discrimination (e.g., Molnar et al., 2014), we found enhanced N100 components for deviant stimuli, as well as late negativities between 400 and 700 ms reminiscent of the ‘late discriminatory negativity’ (e.g., Cheour et al., 2001; Mueller et al., 2008). In our data, both components were more prominent in linguistic conditions, perhaps most compellingly for [ᴧm] stimuli in the learners (**Figure 5B**). Differences in the N100 may be due to stronger habituation when a given stimulus serves as a standard and is frequently repeated. Since [ᴧm] is the only vowel-initial stimulus in our experiment, the onset components (P100, N100, P200) are generally larger than for the other stimuli (in both groups and both conditions; cf. **Figures 3B** and **5B**), rendering such effects more likely. With respect to the observed late negativity in the results from the learner group, a possible interpretation is that this reflects the detection of some physical deviance (acoustic or phonetic), but that this cannot be mapped to a stored phonological representation. Where the MMN reflects automatic processing of sounds in the primary auditory cortex, the late negativities may reflect more controlled processes involving comparisons based on conscious memory of previously heard stimuli (e.g., Cheour et al., 2001; Molnar et al., 2014). In our study, the comparisons may have been made on the basis of estimated duration: though a variable SOA was used to minimize participants’ ability to rely on length differences (apparently successfully so, given the lack of any MMN), the fact remains that [ᴧm] items were shorter than [hᴧm] items. The pattern thus far may be interpreted as additional evidence against the hypothesis that the learners’ difficulties with [h] reflect a general acoustic problem with this segment. Rather, our results suggest that the problem is linguistic in nature: French speakers encounter difficulty with English [h] once they are tasked with constructing and accessing an appropriate phonological representation for this segment.

## Conclusion

The results obtained in this study show that French-speaking learners of English behave much like native English speakers in their perception of [h] in a non-linguistic task in that both groups elicited MMN and P3a components, demonstrating that they were able to detect the presence of [h] on deviant items when these were presented as noise bursts. When [h] was presented as a linguistic stimulus (in a syllable), the two groups behaved differently: while the controls were still able to pre-attentively detect [h] on deviant stimuli (eliciting an MMN), the learners were not. The absence of an MMN in the linguistic condition for the learners is interpreted as indicating that they are unable to construct an appropriate representation for the deviant stimulus: in this condition, the stimuli are clearly linguistic, and therefore require the ability to build and access an appropriate phonological representation for [h]. These results thus constitute neurobiological evidence against the hypothesis that the learners cannot hear English [h] in a strict acoustic sense: though its acoustic properties conspire toward non-salience, native French speakers are able to detect it in a non-linguistic task. Our results suggest that French speakers’ difficulties with [h] lie in its phonological representation in the interlanguage grammar, not in its physical properties.

Further confirmation of our findings would require examination of the phonological representation of [h] in interlanguage grammars. If, as our results suggest, learners encounter difficulty constructing a phonological representation for [h], it may be that they are unable to construct any representation for the segment at all. Probing this requires a task that prompts participants to consult phonological representations as they are stored in lexical entries, and additional investigation of attention and task effects would allow us to tease apart the roles each of these may have in addition to issues of phonological representation in the persistent difficulty that French speakers encounter with [h].

## Author Contributions

JM: task design, data collection, interpretation of results, writing and editing. HG: task design, interpretation of results, writing and editing. KS: task design, data collection, statistical analysis, interpretation of results, writing and editing.

## Conflict of Interest Statement

The authors declare that the research was conducted in the absence of any commercial or financial relationships that could be construed as a potential conflict of interest.

## Footnotes

1 The apostrophe indicates the site of [h]-deletion, not substitution by [ʔ] (Julie Auger, p.c.).

2 A distracter was included to allow us to determine that participants were not responding to differences in overall stimulus length. Items with [𝜃] were used as deviant items in all stimulus blocks; if an elicited MMN reflects detection of greater overall length of [h] items, then the response for [𝜃] items in blocks with a non-[h] standard should be more negative in the time window of the MMN than those obtained in blocks with an [h] item as the standard. While we do not report on the [𝜃] data here, no such difference among these items was observed in preliminary analyses, consistent with the interpretation that any observed MMN is based on presence or absence of [h], and not differences in overall stimuli length.

3 Both the Moroccan and Swiss participants indicated that French was the language they used exclusively in childhood both with family and outside the home. To ensure that passive exposure to Arabic and German respectively (both of which have [h]) did not influence the results of these two participants, the data analysis was also performed with these two participants excluded. The same effects were observed to reach significance, with no new effects emerging; we have therefore included these participants in the results presented here.

4 We thank Reviewer #2 for suggesting these additional analyses with factor ‘Testing Site’ and report them in the Supplementary Materials.

5 The number of trials did not differ across conditions, and the number of trials per subject did not differ between the English and French groups.

6 For effects where *dF* > 1, analysis included Greenhouse–Geisser correction.

7 Note that the ERPs of this contrast at posterior electrodes start to differ much earlier than at other electrodes, within 50 ms after stimulus onset. As discussed in Steinhauer and Drury (2012), sustained ERP differences with onset latencies of less than 100 ms are likely due to baseline problems in data analysis. This may have been an issue with this particular contrast at Pz and may have led to an over-estimation of the negativity at posterior electrodes (in all subsequent time windows). However, since additional analyses with a different baseline compensating for this early difference did not result in major changes (i.e., the sustained posterior negativity was still present, although with a slightly smaller amplitude), and since this negativity is not crucial for the claims made in this paper, we have kept the ERPs as displayed in **Figure 5B** (rather than employing an unconventional post-onset baseline).

8 This pattern in the [ᴧm] condition requires additional explanation. First, **Figure 3B** suggests a (frontal) positivity in the MMN time window for the controls, whereas **Figure 5B** suggests a (posterior) negativity for the learners. One might, therefore, expect some kind of *Match x Group* interaction. Group contrasts limited to the [ᴧm] condition reveal such an interaction (*p* < 0.02). However, in the absence of any interactions with factor *Group* at the higher level of analysis (i.e., for all non-[h] items, shown in **Table 3**), the strict hierarchical approach to ANOVAs recommended in the literature (e.g., Nieuwenhuis et al., 2011) does not allow us to interpret these apparent group differences. The most straightforward interpretation is, therefore, that in both groups, the difference wave showed a gradient of relative negativity at posterior electrodes and of relative positivity at frontal electrodes. Second, as pointed out in discussion of **Figure 5B** (see^7^), the negativity for the learners had a very early onset at posterior electrodes and may be overestimated at those electrodes.

9 L2 learners at very high levels of proficiency showed ERP patterns similar to native speakers. Whether this suggests that even phonological problems may be overcome at very high levels of L2 proficiency cannot be answered with the data at hand (however, see Steinhauer et al. (2009) for native-like ERP patterns in L2 morphosyntactic processing).

10 Note that in written sentences, the learners exhibit an N400 for *curly air* as well.

11 Given that their study took place in Montreal, the native English speaker who produced their stimuli was Canadian and, thus, *a* was low back [a].

## References

[B1] Boersma P. Weenink D. (2010). *Praat: Doing Phonetics by Computer [Computer Program]*, Version 5.1.36. Available at: http://www.praat.org/ (accessed June 24, 2010).

[B2] Cheour M. Korpilahti P. Martynova O. Lang A.-H. (2001). Mismatch negativity and late discriminative negativity in investigating speech perception and learning in children and infants. *Audiol. Neurootol.* 6 2–11. 10.1159/000046804 11173771

[B3] Davis S. Cho M.-H. (2003). The distribution of aspirated stops and /h/ in American English and Korean: an alignment approach with typological implications. *Linguistics* 41 607–652. 10.1515/ling.2003.020

[B4] Dogil G. (1988). “Phonological configurations: natural classes, sonority and syllabicity,” in *Features, Segmental Structure, and Harmony Processes*, eds van der Hulst H. Smith N. (Dordrecht: Foris), 79–103.

[B5] Eulitz C. Lahiri A. (2004). Neurobiological evidence for abstract phonological representations in the mental lexicon during speech recognition. *J. Cogn. Neurosci.* 16 577–583. 10.1162/089892904323057308 15185677

[B6] Janda R. D. Auger J. (1992). Quantitative evidence, qualitative hypercorrection, sociolinguistic variables – and French speakers’ ‘eadhaches with English h/Ø. *Lang. Commun.* 12 195–236. 10.1016/0271-5309(92)90015-2

[B7] John P. (2006). *Variable h-epenthesis in the Interlanguage of Francophone ESL Learners*. MA thesis, Concordia University, Montreal, QC.

[B8] Keating P. (1988). Underspecification in phonetics. *Phonology* 5 275–292. 10.1017/S095267570000230X

[B9] LaCharité D. Prévost P. (1999). “The role of L1 and teaching in the acquisition of English sounds by francophones,” in *Proceedings of BUCLD 23*, eds Greenhill A. Littlefield H. Tano C. (Somerville, MA: Cascadilla Press), 373–385.

[B10] Ladefoged P. (2001). *A Course in Phonetics*, 4th Edn. Oxford: Blackwell.

[B11] Ladefoged P. Maddieson I. (1996). *The Sounds of the World’s Languages*. Oxford: Blackwell.

[B12] Lahiri A. Reetz H. (2002). “Underspecified recognition,” in *Labphon 7*, eds Gussenhoven C. Werner N. Rietveld T. (Berlin: Mouton), 637–676.

[B13] McCarthy J. J. (1988). Feature geometry and dependency: a review. *Phonetica* 43 84–108. 10.1159/000261820

[B14] McCarthy J. J. (1994). “The phonetics and phonology of Semitic pharyngeals,” in *Papers in Laboratory Phonology III*, ed. Keating P. (Cambridge: Cambridge University Press), 191–233.

[B15] Molnar M. Polka L. Baum S. Steinhauer K. (2014). Learning two languages from birth shapes pre-attentive processing of vowel categories: electrophysiological correlates of vowel discrimination in monolinguals and simultaneous bilinguals. *Biling. Lang. Cogn.* 17 526–541. 10.1017/S136672891300062X

[B16] Mueller V. Brehmer Y. von Oertzen T. Li S.-C. Lindenberger U. (2008). Electrophysiological correlates of selective attention: a lifespan comparison. *BMC Neurosci.* 9:18 10.1186/1471-2202-9-18 PMC2270855 18237433

[B17] Näätänen R. (1999). The perception of speech sounds by the human brain as reflected by the mismatch negativity (MMN) and its magnetic equivalent (MMNm). *Psychophysiology* 38 1–21. 10.1111/1469-8986.3810001 11321610

[B18] Näätänen R. Paavilainen P. Rinne T. Alho K. (2007). The mismatch negativity (MMN) in basic research of central auditory processing: a review. *Clin. Neurophysiol.* 118 2544–2590. 10.1016/j.clinph.2007.04.026 17931964

[B19] Narayanan S. Alwan A. Haker K. (1995). An articulatory study of fricative consonants using magnetic resonance imaging. *J. Acoust. Soc. Am.* 98 1325–1347. 10.1121/1.413469

[B20] Nieuwenhuis S. Forstmann B. U. Wagenmakers E.-J. (2011). Erroneous analyses of interactions in neuroscience: a problem of significance. *Nat. Neurosci.* 14 1105–1107. 10.1038/nn.2886 21878926

[B21] Phillips C. Pellathy T. Marantz A. Yellin E. Wexler K. Poeppel D. (2000). Auditory cortex accesses phonological categories: an MEG mismatch study. *J. Cogn. Neurosci.* 12 1038–1055. 10.1162/08989290051137567 11177423

[B22] Rose S. (1996). Variable laryngeals and vowel lowering. *Phonology* 13 73–117. 10.1017/S0952675700000191

[B23] Schluter K. Politzer-Ahles S. Almeida D. (2016). No place for /h: an ERP investigation of English fricative place features. *Lang. Cogn. Neurosci.* 31 728–740. 27366758 10.1080/23273798.2016.1151058 PMC4917926

[B24] Sharma A. Dorman M. F. (1999). Cortical auditory evoked potential correlates of categorical perception of voice-onset time. *J. Acoust. Soc. Am.* 106 1078–1083. 10.1121/1.428048 10462812

[B25] Sokolov E. N. Spinks J. A. Näätänen R. Lyytinen H. (2002). *The Orienting Response in Information Processing*. Mahwah, NJ: Lawrence Erlbaum, 115–117.

[B26] Steinhauer K. Drury J. E. (2012). On the early left-anterior negativity (ELAN) in syntax studies. *Brain Lang.* 120 135–162. 10.1016/j.bandl.2011.07.001 21924483

[B27] Steinhauer K. White E. Drury J. E. (2009). Temporal dynamics of late second language acquisition: evidence from event-related brain potentials. *Second Lang. Res.* 25 13–41. 10.1177/0267658308098995

[B28] Steriade D. (1987). “Locality conditions and feature geometry,” in *Proceedings of NELS 17*, eds McDonough J. Plunkett B. (Somerville, MA: Cascadilla), 595–617.

[B29] Strange W. (2011). Automatic selective perception (ASP) of first and second language speech: a working model. *J. Phon.* 39 456–466. 10.1016/j.wocn.2010.09.001

[B30] Sussman E. S. (2007). A new view on the MMN and attention debate: the role of context in processing auditory events. *J. Psychophysiol.* 21 164–175. 10.1027/0269-8803.21.34.164

[B31] Walker D. C. (2001). *French Sound Structure.* Calgary, AB: University of Calgary Press.

[B32] Werker J. F. Tees R. C. (1984). Phonemic and phonetic factors in adult cross-language speech perception. *J. Acoust. Soc. Am.* 75 1866–1878. 10.1121/1.390988 6747097

[B33] White E. J. Titone D. Genesee F. Steinhauer K. (2015). Proficiency, task, and stimulus effects reflected in ERP correlates of phonetic perception by late second language learners. *Biling. Lang. Cogn.* 1–22. 10.1017/S1366728915000620

