# Supplementary material for: Using Event-Related Brain Potentials to Assess Perceptibility: The Case of French Speakers and English [h]
Source: Front Psychol. 2016 Oct 4;7:1469. doi: 10.3389/fpsyg.2016.01469 (PMC5048474; doi:10.3389/fpsyg.2016.01469)
Supplement: Supplementary file 1 [file Data_Sheet_1.pdf]

Supplementary Material

Using event-related brain potentials to assess perceptibility: The case of French speakers and English [h]

Jennifer Mah*, Heather Goad, Karsten Steinhauer*

*** Correspondence:** Corresponding Authors: jmah@mtroyal.ca, karsten.steinhauer@mcgill.ca

1 Supplementary Data

Analyses with factor 'Testing Site':

Given that some of our (collapsed) data in both groups were collected in Montreal and others in Calgary, it was possible that the different locations might have led to different ERP patterns (e.g., due to different EEG systems). Especially for the group of French learners, it was also possible that slight differences in English proficiency could have led to distinct patterns of processing. Since our experimental paradigm was an unattended oddball and did not generate behavioural performance data that could be used to test this hypothesis, we re-ran our ERP analyses and included the factor 'Testing Site' (Montreal vs. Calgary) in our ANOVAs. We thank Reviewer 2 for suggesting these additional analyses. In summary, they did not reveal any important influence of factor 'Testing Site' on our data or their interpretation. Below we briefly report the findings for all ERP components (MMN, P3a, N100, late negativity); we emphasize where significant interactions with this factor were found, and what the corresponding follow-up analyses revealed about the underlying pattern.

1.1 Mismatch negativity:

When we included factor 'Testing Site' in the global ANOVA for the MMN, all original effects reported in our paper (see our original Table 2) were replicated, while none of these effects interacted with factor 'Testing Site' (see **new Table 2A below**). Moreover, there was not a single significant interaction involving both factor 'Group' (English native speakers vs. French learners) and factor 'Testing Site', indicating that the group differences reported in our article were robust, irrespective of where the participants were tested. Most importantly for our main claims regarding the MMN, the 4-way interaction *Match x H-presence x Type x Group* again reached significance, without any indication of an influence of factor 'Testing Site' (F = 0.04).

Table 2A: Global ANOVA (MMN) for Group comparisons and within group follow-ups.

Effect Source	dF	Group Contrast (n=41)		Controls (n=24)		Learners (n=17)	
		F-value	*p*-value	*F*-value	*p*-value	*F*-value	*p*-value
Match	1	**11.31**	**0.0018**	2.67	0.1158	7.06	0.0172
Match x Site	1	2.04	0.1618				
Match x H-presence	1	**13.25**	**0.0008**	10.01	0.0043	2.21	0.1563
Match x H-pres x Site	1	3.78	0.0592				
Match x H-presence x Electrode	2	**15.40**	**<0.0001**	12.8	0.0002	5.12	0.0208
Match x Type x Elec x Site	2	0.06	0.9072				
Match x Type x Electrode	2	**7.80**	**0.0033**	5.73	0.0162	4.28	0.0334
Match x Type x Elec x Site	2	1.37	0.2570				
Match x H-presence x Type x Group	1	**4.22**	**0.0471**				
Match x H-pres x Type x Group x Site	1	0.04	0.8463				

Even though the global ANOVA above did not point to any substantial influence of factor 'Testing Site' on our MMN findings, we also re-ran the contrasts for conditions 'with [h]' versus 'without [h]' (underlying our original Table 3), for two reasons. First, these analyses were critical in establishing the relevant group differences reported in our article. Secondly, Table 2A above included one single interaction with factor 'Testing Site' that approached significance: ***Match * H-pres * Site*** (p = 0.0592), thus potentially pointing to different patterns in Calgary versus Montreal.

Table 3A summarizes the results. In general, all significant effects from our original Table 3 were replicated, and only one of them – the strong **main effect of *Match* in the Group contrast 'with [h]' – interacted with factor 'Testing Site' (p = 0.0464)**, thereby also accounting for the marginal *Match * H-pres * Site* interaction in Table 2A.

However, follow-up analyses for each testing site showed that **both the participants tested in Montreal (F(1,23) = 5.71; p = 0.0254) and those tested in Calgary (F(1,14) = 10.08; p = 0.0068)**

did show a significant *Match* effect; it was simply somewhat stronger in the latter group. This also explains why the main effect of *Match* across both testing sites ($F(1,38) = 18.94$, $p < 0.0001$) was much more significant than the interaction with 'Site'.

Most importantly for our group differences, the *Match x Type x Group* interaction still reached significance ($p = 0.0227$) and was not influenced by factor 'Testing Site' ($p > 0.26$).

In sum, we found no evidence that our MMN data were influenced by factor 'Testing Site'.

Table 3A: ANOVAs (MMN) separately for [h]-presence and [h]-absence.

Effect	Group Contrast with [h]		Controls with [h]		Learners with [h]		Group Contrast no [h]	
Source	*F*-value	*p*-value	*F*-value	*p*-value	*F*-value	*p*-value	*F*-value	*p*-value
Match	**18.94**	**0.0001**	7.8	0.0103	8.7	0.0094	0.28	0.6001
Match x Site	**4.25**	**0.0464**					0.04	0.8517
Match x Electrode	**6.98**	**0.0017**	2.57	0.1068	4.33	0.0444	7.24	0.0013
Match x Elec x Site	0.27	0.7662					0.09	0.9135
Match x Type x Group	**5.66**	**0.0227**	-	-	-	-		
Match x Type x Group x Site	1.31	0.2604						
Match x Type			0.01	0.9266	**8.61**	**0.0097**		
Match x Type x Electrode							7.14	0.0050
Match x Type x Elec x Site							3.45	0.0554

1.2 P3a component:

Similar to our MMN analyses, re-running the global ANOVA for the P3a time window with factor 'Testing Site' replicated all effects reported in our article with only minor changes in the F- and p-values:

H-presence x Match (p-value was 0.0245, now: 0.0238);

Type x Match (p-value was 0.0001, now: 0.0001);

H-presence x Type x Match (p-value was 0.0022, now: 0.0030);

H-presence x Type x Match x Electrode (p-value was 0.0172, now: 0.040).

Of these effects, only the 4-way interaction also interacted with factor 'Testing Site' (*H-presence x Type x Match x Electrode x Site:* $F(2,74) = 4.45$, $p = 0.0285$), suggesting slightly different scalp topographies of the P3a effect in Montreal compared to Calgary. However, since not a single interaction with factor group was found, our conclusion regarding the P3a remains unaffected: Both groups (i.e., English native speakers and French learners) displayed the exact same pattern, in that a P3a was only observed for the non-linguistic [h] condition (i.e., for [hf]), whereas no P3a was found in any other condition (including the linguistic [hʌm] items).

1.3 N100 component:

While the *Type x Match* effect reported for the N100 did not interact with factor 'Testing Site' ($F(1,37) = 0.75$; $p = 0.3936$), the inclusion of factor 'Testing Site' in this ANOVA slightly reduced the F-value for the *Type x Match* effect itself. In the analyses reported in our paper, the effect reached significance ($F(1,39) = 5.01$; $p = 0.031$), whereas in the modified ANOVA it just failed to reach significance ($F(1,37) = 4.04$, $p = 0.0519$).

We believe that the new analyses do not justify any changes in the article, for two reasons. First, even after inclusion of factor 'Testing Site', this effect is very close to reaching significance. Secondly, given that the F-value for the 3-way interaction *Type x Match x Site* is smaller than 1.0, there really is no evidence for any impact of the testing site. Therefore, including this factor does not improve the model, and our original analysis should be viewed as the more appropriate one.

1. 4 Late negativity:

Inclusion of factor 'Testing Site' resulted in the same effects reported in the paper. None of these effects was significantly influenced by factor 'Testing Site'.
